# Supplementary material for: High flavivirus structural plasticity demonstrated by a non-spherical morphological variant
Source: Nat Commun. 2020 Jun 19;11:3112. doi: 10.1038/s41467-020-16925-y (PMC7305169; doi:10.1038/s41467-020-16925-y)
Supplement: Supplementary file 1 — Supplementary Information [file 41467_2020_16925_MOESM1_ESM.pdf]

Supplementary Information

High flavivirus structural plasticity demonstrated by a non-spherical morphological variant

Morrone et al.

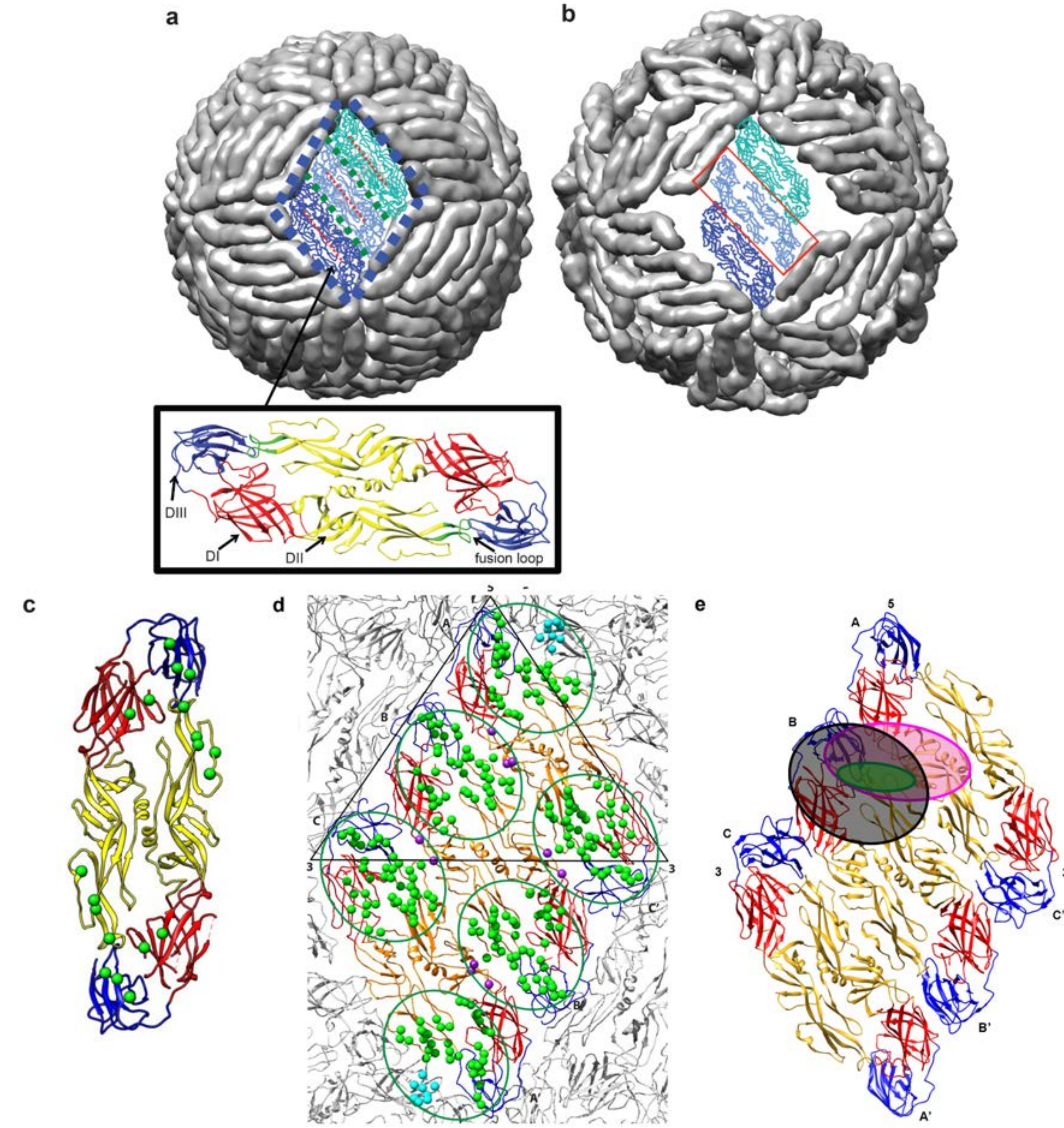

Supplementary Figure 1. The organization of E proteins on the icosahedral spherical flavivirus particles. **a** The structure of the smooth-surfaced spherical mature flavivirus particle (*13*) (left). E proteins are packed tightly and they exist as dimers. Three E protein dimers lie parallel to each other forming a raft and there are 30 rafts arranged in a herringbone pattern on the virus surface. The intra-E, inter-E, and inter-raft E protein interfaces are indicated by dotted red, green and blue lines, respectively. One of the rafts is shown in wires, and the three dimers within that raft are colored in shades of blue. The zoom-in view of an E protein dimer (boxed below) shows the E ectodomain colored according to their domains (red = DI, yellow = DII, blue = DIII), and the fusion loop is colored in green. Fusion loops in the spherical smooth-surfaced particles are tucked between DI-DIII regions of the opposite E protein protomer. **b** The structure of the bumpy-surfaced spherical

mature DENV2 (*3*) (PDB: 3ZKO), showing looser E protein organization compared to the compact surfaced spherical particle in (a). The separation of the E proteins within the dimer at the icosahedral 2f vertex is observed (red box). **c** The crystal structure of Fab C10 bound to the DENV2 ectodomain E protein dimer (*17*), showing the epitope bound by Fab C10 (green spheres). **d** The epitope of Fab C10 on the E protein raft of the cryoEM Fab C10:ZIKV smooth-surfaced spherical particle structure (*13*). An asymmetric unit is presented by a black triangle, with symmetry vertices indicated. Residues which interact with Fab C10 are presented as spheres: cyan spheres indicating inter-raft residues and purple spheres as inter-dimer residues; intra-dimer residues are colored in green. The region forming one epitope is circled in green. In the cryoEM structure of Fab C10:ZIKV smooth-surfaced spherical particle structure (*13*), at low pH the Fab interactions with the E proteins at the inter-raft interface is disrupted, whereas those at the intra-dimer and inter-dimer stayed intact suggesting the Fab binding to the inter-raft E interface is weaker than at the other interfaces. **e** Epitopes bound by antibodies C10, 2A10G6 (fusion loop), and 5J7 on the E proteins inside a raft are circled and shaded in black, green and pink, respectively.

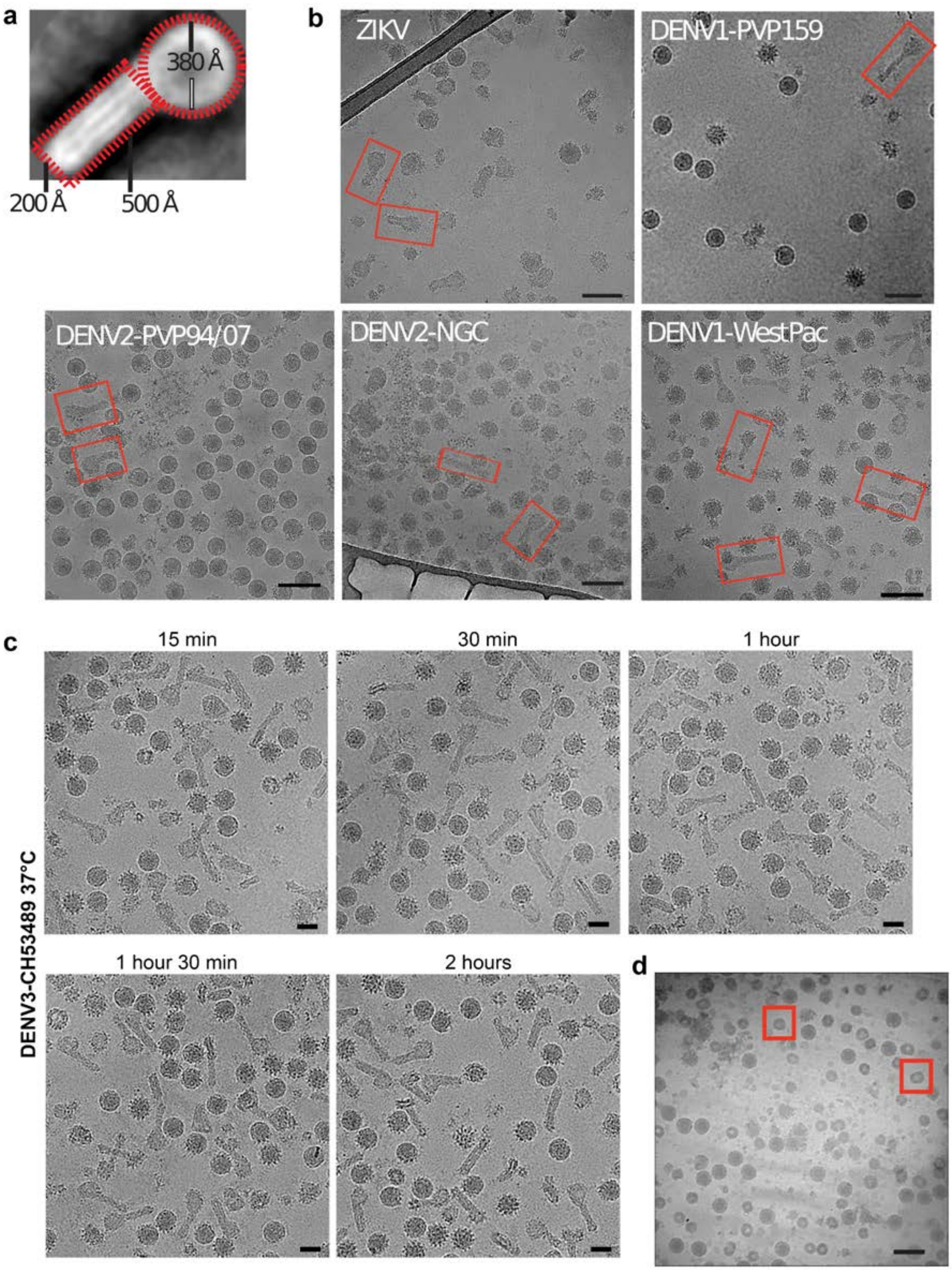

Supplementary Figure 2. Particles of DENV serotypes and strains, and ZIKV are observed to be able to form clubSPs, although they represent a minority of the virus population.

a A 2D class-average of DENV3-CH53489 clubSPs at 37 °C. The head and tail are outlined in a circle and rectangle and their measurements are shown. Data

represents one of fifteen independent experiments. **b** Respresentaitve micrographs of various DENV serotypes and strains, and ZIKV showed presence of clubSPs (representative in each boxed in red). Scale bars on micrographs represent 100 nm. n=3 independent experiments. **c** Micrographs of DENV3-CH53489 incubated at 37°C for different lengths of time (15min, 30min, 1 hour, 1.5 hour and 2 hours). Scale bar represents 50nm. n=1. **d** Micrograph of DENV2-PVP94/07 strain showing some particles have an empty or partially filled core (representative is boxed in red). Their centers are much lower in intensity than the full particles. Scale bar on micrographs represent 100 nm. n=1.

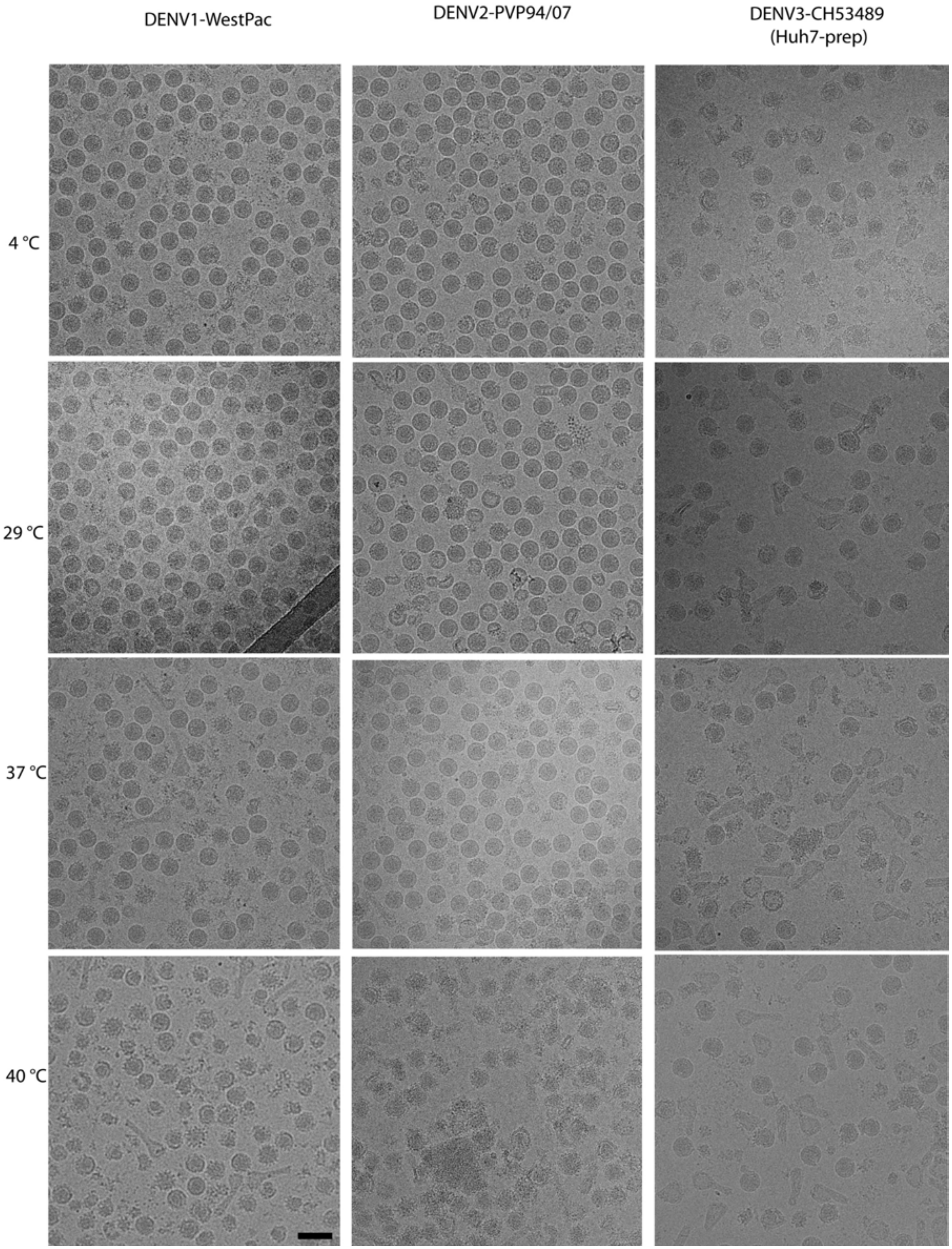

Supplementary Figure 3. ClubSPs form in multiple strains at the same temperature range. Shown are three different serotypes of DENV incubated at various temperatures (DENV3 in mammalian cells), showing that the clubSPs form at temperatures higher than 4 °C. Scale bar represents 100 nm. n=3 (DENV1-

Westpac, n=3 (DENV2-PVP94/07 and n=1 (DENV3-CH53489 ; Huh-7 prep) respectively.

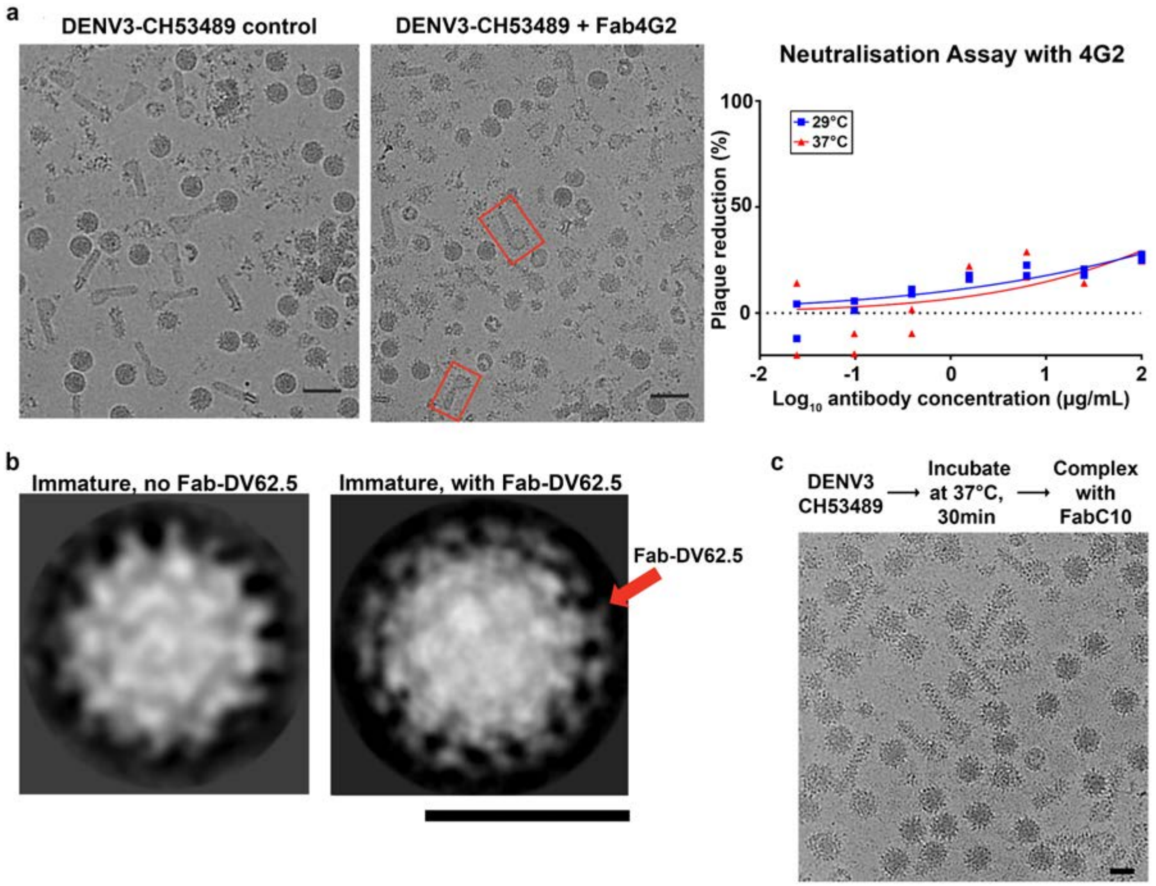

Supplementary Figure 4. Particles of DENV3-CH53489 when complexed with different Fabs.

a Micrographs of DENV3-CH53489 incubated at 29 °C; left is the negative control, center is incubation with Fab-4G2 at 29 °C for 30 min after pre-incubation. Two spiky clubSPs are boxed in red for example. Scale bar represents 100nm. Right panel is the neutralization profile of antibody 4G2 to DENV3-CH53489 clubSP induced at 29 °C and 37 °C. Antibody 4G2 is unable to neutralize the virus at all concentrations tested. Neutralisation curve represents best fit from the average of two independent experiments. Representative data from two independent experiments. Source data are provided in the source data file. **b** The 2D class averages of uncomplexed immature spherical DENV3-CH53489 particles (left), and the Fab-DV62.5 bound complex (right). The extra densities corresponding to Fab bound on the viral surface are indicated by red arrow. Scale bar represents 50nm. n=1. **c**. Micrograph of DENV3-CH53489 incubated at 37°C for 30min before addition of Fab C10. Scale bar represents 50nm. n=1.

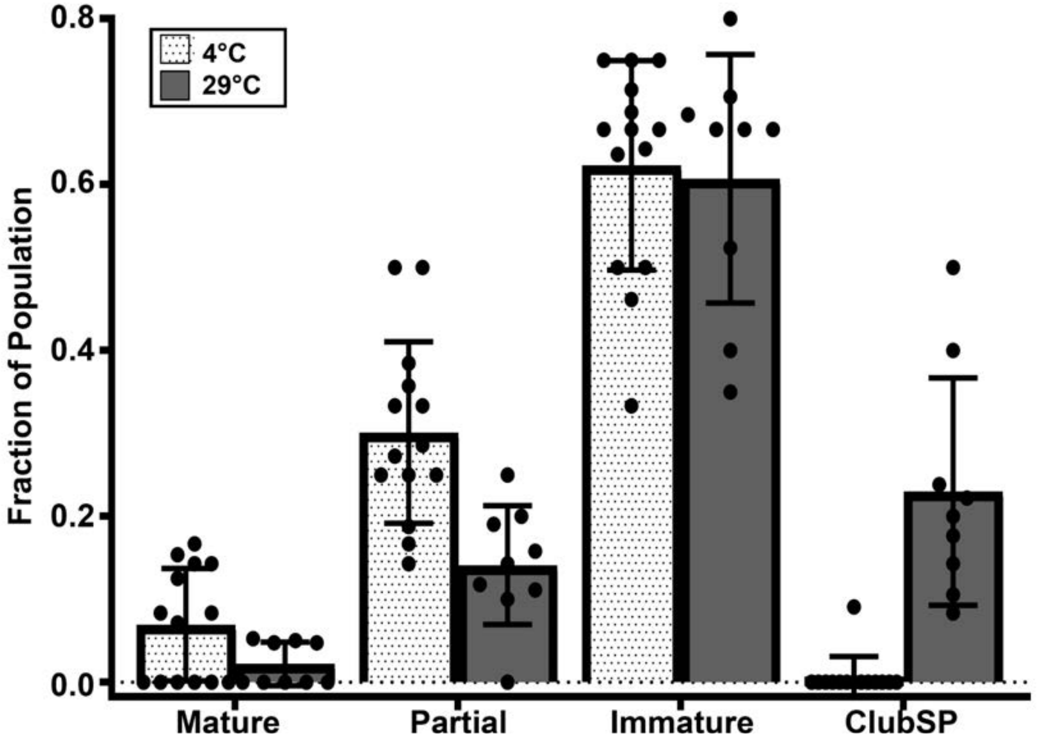

Supplementary Figure 5. DENV3-CH53489 are not formed from highly immature particles.

Population counts of particles (fully mature, partially immature, highly immature and clubSP particles) from an immature virus DENV3-CH53489 preparation, made by growing virus-infected cells in the presence of ammonium chloride. When immature virus is incubated at 29 °C (the temperature that induces clubhead particles formation in the mature virus prep), an increase in the number of clubSP particles was observed. The number of immature virus does not change at 29 °C compared to 4 °C whereas the mature and partially immature virus count showed a decrease. This suggests that mature and partially immature virus at 4 °C may have changed to clubSP structure at 29 °C and also the clubhead particles likely do not originate from the highly immature virus particles. Data are presented as mean with error bar represents SD from at least nine micrographs. Source data are provided in the soure data file.

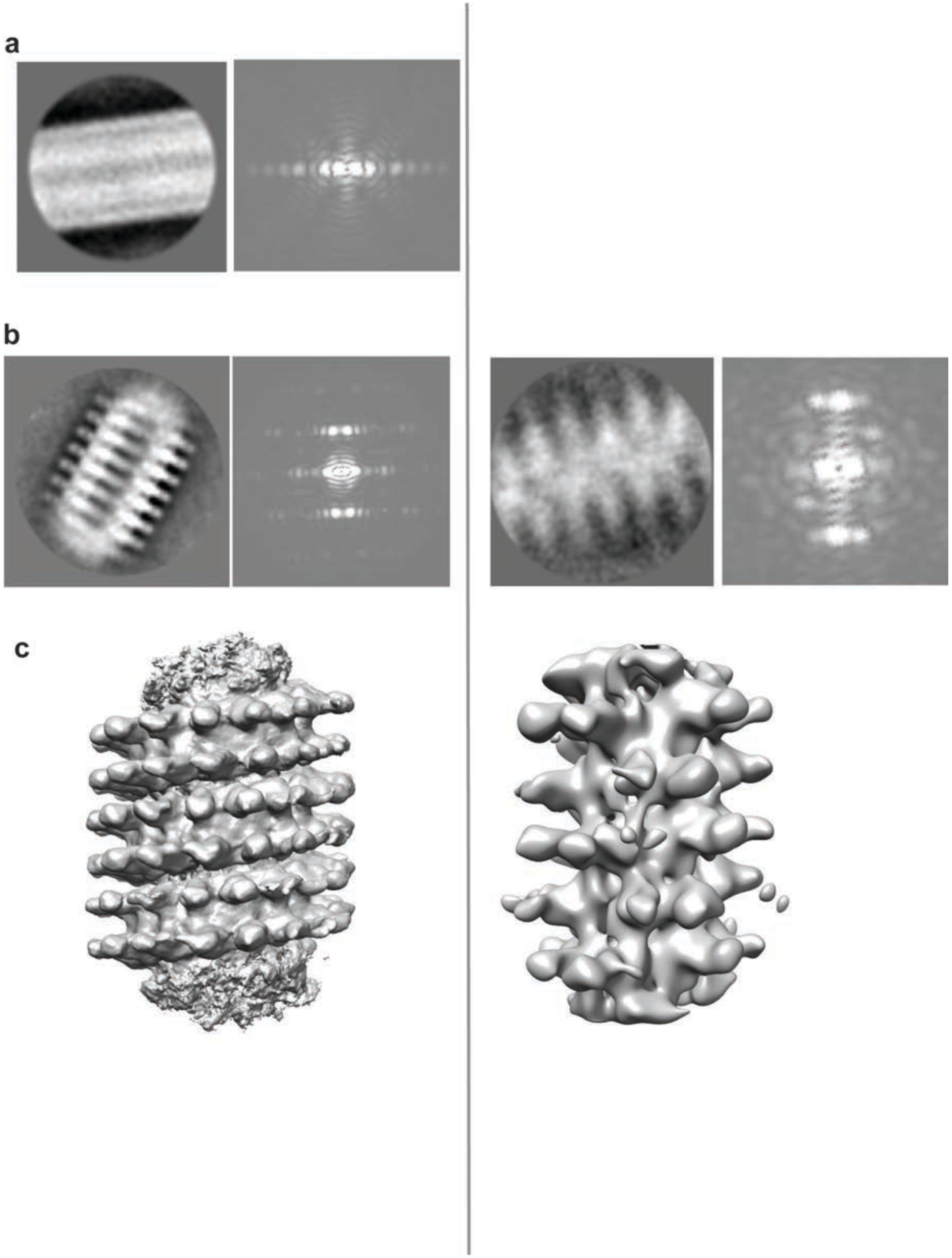

Supplementary Figure 6. Asymmetric reconstructions of the central segments of the tail of C10:DENV3 clubSPs and the whole of C10:ZIKV catSPs are used to determine the initial helical parameters.

a 2D-class average of uncomplexed DENV3-CH53489 clubSP (left) and its power spectrum (right). The smooth surface seen in the class average and the presence of only one layer line in the power spectrum suggests the structure cannot be solved by single particle reconstruction or helical reconstruction. **b** In contrast, when Fab C10 is bound to ZIKV (left) or the tail of DENV3-CH53489 (right), the power-spectra of the boxed filaments showed presence of multiple strong layer lines indicating presence of helical symmetries. **c** Initial asymmetric cryoEM map of the Fab C10:ZIKV catSP (left) and Fab C10:DENV3-CH53489 (right). Densities corresponding to the Fabs C10 are clearly observed, allowing fitting of the structure of E protein dimer complexed with C10 to derive the initial helical parameters.

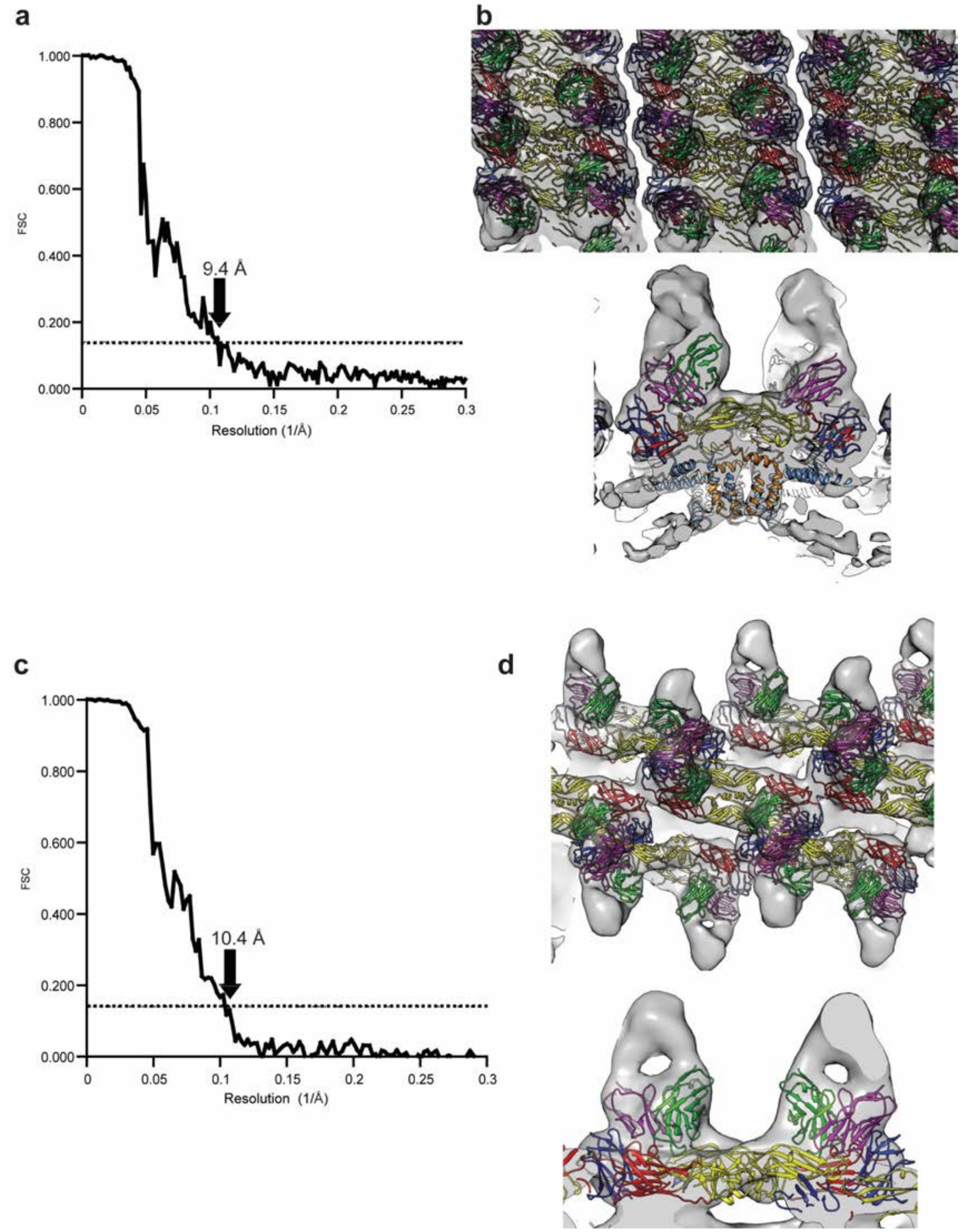

Supplementary Figure 7. The helical reconstructions of tail central segments of the C10:DENV3 clubSPs, and central portion of the C10:ZIKV catSPs.

a The FSC curve of the helical reconstruction cryoEM map of Fab C10:ZIKV catSP. **b** Top (top panel) and side views (bottom panel) of the Fab C10:ZIKV E protein dimer (PDB 5H37) into the densities of the C10:ZIKV CatSP helical map (grey). The E protein dimer bound Fab C10 are shown as ribbon and colored as follows: E-DI

(red), E-DII (yellow), E-DIII (blue), E-transmembrane (light-blue), M-protein (orange), Fab-C10 heavy chain (magenta) and light chain (green). The density of the transmembrane region, including the M protein, is clearly resolved (bottom panel). **c**, A plot of Fab C10:DENV3-CH53489 FSC curve. **d**, The fit of the homology model of Fab C10:DENV3-CH53489 E protein dimer into the helical cryoEM map of C10:DENV3-CH53489. Due to the poorer resolution (10.4 Å resolution) the transmembrane regions are not fitted. For (a,c), the FSC 0.143 cutoff is shown as a dotted line. The resolutions reported are shown as black arrows.

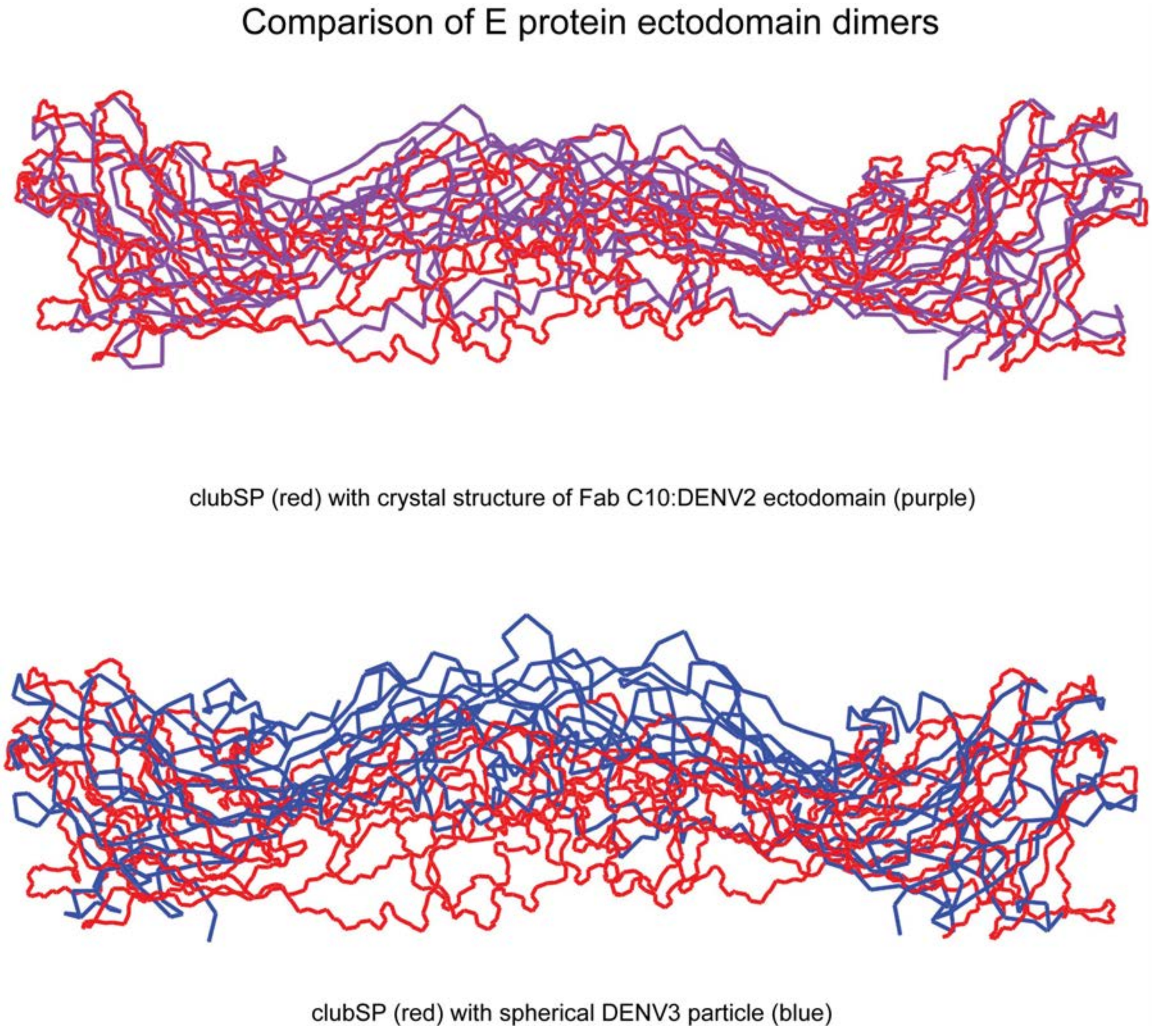

Supplementary Figure 8. Superposition of the E ectodomain (red) of the Fab C10:DENV3-CH53489 clubSP structure with that of the crystal structure of Fab C10:DENV2 ***(17)*** **(purple, in top panel), and the cryoEM structure of the spherical DENV3 virus particle** ***(12)*** **(blue in bottom panel) suggests the curvature of the E protein dimer in the clubSP structure is more similar to the recombinant E protein structure (top panel).**

	**Fab C10:DENV3-CH53489 complex (EMDB 30278) (PDB 7C2S)**	**Fab C10-:ZIKV complex (EMDB 30279) (PDB 7C2T)**
**Data collection and processing**		
Magnification	47,000	59,000
Voltage (kV)	300	300
Electron exposure ($e–/Å^2$)	18	18
Defocus range (μm)	Underfocus 0.6 – 4.8	Underfocus 0.5 – 2.5
Pixel size (Å)	1.71	1.34
Symmetry imposed	Helical<br>rise 18.5 Å<br>twist 102.3°	Helical<br>rise 8.6 Å<br>twist 24.1°
Initial particle images (no.)	9006 segments	3936 segments
Final particle images (no.)	7051 segments	3406 segments
Map resolution (Å)<br>FSC threshold 0.143	10.4	9.4
Map resolution range (Å)	60 – 10.4	60 – 9.4
**Refinement**		
Initial model used (PDB code)	1UZG additionally modified with SWISS-MODEL	5H37
Model resolution (Å)	17.26	10.44
FSC threshold	0.5	0.5
Model resolution range (Å)	N/A	N/A
Map sharpening *B* factor ($Å^2$)	-705	-286
Model composition<br>Non-hydrogen atoms<br>Protein residues<br>Ligands	1220 protein residues	1630 protein residues
B factors ($Å^2$)<br>Protein<br>Ligand	20	20
R.m.s. deviations<br>Bond lengths (Å)<br>Bond angles (°)	N/A	N/A
Validation<br>MolProbity score<br>Clashscore<br>Poor rotamers (%)	N/A	N/A
Ramachandran plot<br>Favored (%)<br>Allowed (%)<br>Disallowed (%)	N/A	N/A

Supplementary Table 1. Cryo-EM data collection, refinement and validation statistics

Particle	Inner radius (Å)	Inner length (Å)	Cylindrical volume (m^3)	Spherical volume (m^3)	Copies of Viral Genomes allowed*
DENV and ZIKV Spherical round particles	150			1.4E-23	3.7
DENV3 clubSP head	140	120	7.4E-24		1.9
Fab C10:ZIKV catSP	75	450	7.9E-24		2.1

*based on 11,000 nucleotides, with average nucleotide volume calculated by legend reference.

Supplementary Table 2. DENV3 clubSPs and ZIKV catSPs can accommodate viral RNA genome.

Calculation of volumes of spherical virus particles assuming a spherical shape, and the head of clubSP and the whole catSP assuming cylindrical shape, based on measurements done on 2D class-averages or electron density maps of inner volumes, with RNA volume measurements based on (*28*). One copy of viral genome is assumed to have a volume of $3.8x10^{-24}$ m^3.
